# Brain-Derived Neurotrophic Factor and Nerve Growth Factor Therapeutics for Brain Injury: The Current Translational Challenges in Preclinical and Clinical Research

**DOI:** 10.1155/2022/3889300

**Published:** 2022-03-02

**Authors:** Serena-Kaye Sims, Brynna Wilken-Resman, Crystal J. Smith, Ashley Mitchell, Lilly McGonegal, Catrina Sims-Robinson

**Affiliations:** ^1^Medical University of South Carolina, 173 Ashley Ave, Charleston, SC 29424, USA; ^2^College of Charleston, 66 George Street, Charleston, SC 29424, USA

## Abstract

Ischemic stroke and traumatic brain injury (TBI) are among the leading causes of death and disability worldwide with impairments ranging from mild to severe. Many therapies are aimed at improving functional and cognitive recovery by targeting neural repair but have encountered issues involving efficacy and drug delivery. As a result, therapeutic options for patients are sparse. Neurotrophic factors are one of the key mediators of neural plasticity and functional recovery. Neurotrophic factors such as brain-derived neurotrophic factor (BDNF) and nerve growth factor (NGF) serve as potential therapeutic options to increase neural repair and recovery as they promote neuroprotection and regeneration. BDNF and NGF have demonstrated the ability to improve functional recovery in preclinical and to a lesser extent clinical studies. Direct and indirect methods to increase levels of neurotrophic factors in animal models have been successful in improving postinjury outcome measures. However, the translation of these studies into clinical trials has been limited. Preclinical experiments have largely failed to result in significant impacts in clinical research. This review will focus on the administration of these neurotrophic factors in preclinical and clinical stroke and TBI and the challenges in translating these therapies from the bench to the clinic.

## 1. Introduction

Ischemic stroke, a leading cause of disability worldwide, results from limited blood flow to the brain due to the blockage or narrowing of arteries. Unfortunately, there are a lack of therapeutic options that can effectively minimize damage or aid in recovery following brain injury from ischemic stroke. The pharmacologic standard of care involves clot breakdown with the thrombolytic agent tissue plasminogen activator (tPA) and/or prevention of further ischemia using anticoagulants such as aspirin. After a stroke, tPA is often the best available option for patients but must be administered within the first 3 hours, or potentially up to 4.5 hours [1]. The short time window in which tPA can be administered, combined with potential complications such as intracranial hemorrhage, has greatly limited its use in some patients [2]. While clot thrombolysis and prevention can be useful in preventing further ischemic damage, by the time a stroke patient receives these treatments, brain injury has already occurred. Despite a need for new stroke treatments, there has been little success in identifying therapeutics that can be widely used to promote neural repair and improve functional recovery following brain injury. Many promising experimental treatments have failed to deliver positive results in clinical trials [3] for reasons including lack of efficacy and target validation and pharmacokinetic and pharmacodynamic issues [4].

One avenue of stroke research has involved exploring the use of neurotrophin treatments as a mechanism for neural repair and enhanced recovery. Neurotrophins are a family of growth factors that play important roles in the survival and function of neurons. There are four known members of the neurotrophin family of growth factors in mammals: brain-derived neurotrophic factor (BDNF), nerve growth factor (NGF), neurotrophin-3 (NT-3), and neurotrophin 4 (NT-4) [5]. Neurotrophins regulate development in the central and peripheral nervous systems by interacting with tropomyosin receptor kinase (Trk) receptors [6]. NGF preferentially binds with TrkA receptors, BDNF and NT-4 with TrkB receptors, and NT-3 with TrkC receptors [5]. The dimerization and autophosphorylation of Trk receptors activate major signaling pathways including PLC-gamma, MAPK/ERK, and PI3K/Akt which suppress apoptosis through their downstream mediators CREB, BCL2, and BAX and Bad, respectively [7]. BDNF and NGF increase the phosphorylation of synapsin 1 for synaptic vesicle release [8]. These four neurotrophins also bind to their low-affinity receptor, p75^NTR^, which induces apoptosis, and in some cases may promote neuronal survival and neurite growth during neurodevelopment [9]. Therefore, neurotrophins are key mediators in neural plasticity postinjury to promote neuronal growth and survival [10]. Of these four neurotrophins, only two, BDNF and NGF, are well studied as potential stroke treatments.

This review will outline the current state of preclinical and clinical research surrounding the potential for BDNF and NGF to become treatment options for patients following stroke. These neurotrophins promote neuroprotection and regeneration and have been examined to determine their role in neural repair as well as their ability to improve functional recovery in preclinical and, to a lesser extent, clinical studies. Direct and indirect methods of increasing levels of these neurotrophins in animal models have demonstrated promise in improving outcome measures following brain injury. Unfortunately, the translation of these preclinical studies into clinical trials has been limited. This review will focus on both direct administration of exogenous neurotrophic factors and indirect methods of modifying endogenous neurotrophic factor levels in the central nervous system after stroke, and it will also examine the challenges involved in moving BDNF and NGF-related treatments from the bench to the clinic.

### 1.1. Challenges with Treatments

Currently, a need exists for the development of additional therapies that are effective for improving recovery from ischemic injury. While there have been promising advances in preclinical studies, many of these therapies have failed to translate clinically. There are several challenges that may contribute to this lack of translation. First, many treatments have poor pharmacokinetic profiles [11]. Second, the presence of the blood-brain barrier limits the ability of many systemically administered therapeutics to access the central nervous system. Finally, preclinical studies vary widely in their methods, and their outcomes have not converged on a consensus as to the effects of neurotrophin treatment or the extent of those effects. These issues may contribute to the challenges in translating preclinical studies into clinical trials. Human trials involving the administration of neurotrophins for ischemic injury are rare, and there have been no large, comprehensive clinical trials. As a result, there is not sufficient information to assess whether current preclinical models and methodologies are adequate to forecast human outcomes.

### 1.2. Poor Pharmacokinetics (ex., Size and Half-Life)

Neurotrophins can form protein-antibody complexes which may affect their tissue distribution, metabolism, and elimination. Additionally, peptidases and proteases in the blood can degrade neurotrophins, leading to reduced bioavailability, as evidenced by poor tissue distribution and short half-lives. An increased dose would be required to compensate for its poor bioavailability. This, in turn, can trigger adverse effects. Administration of neurotrophins can cause immunogenicity which can manifest in adverse effects including hypersensitivity and anaphylactic shock [12]. In order to overcome these pharmacokinetic issues, neurotrophins have been incorporated in drug delivery systems and neurotrophin mimetics with more favorable pharmacokinetics have been developed. Studies involving the implantation of BDNF polymers in the hippocampi of rats indicated that microspheres released the majority of the encapsulated BDNF within 48 hours [13]. Mimetics, which mimic the BDNF protein, were similarly created in an attempt to circumvent the pharmacokinetic issues [14]. The development of nonpeptide molecules that can activate the TrkB receptor without activating the harmful p75NTR receptor has been the subject of current research. *In vitro* experiments demonstrate that the molecule LM22A-4, a selective small-molecule partial agonist of TrkB, can trigger the downstream activators of the TrkB receptor [9]. Although these interventions are still in the preclinical stage, their potential to overcome pharmacokinetic challenges may lead to future use in clinical trials. An additional benefit of mimetics is that they may be better able to cross the blood-brain barrier compared to BDNF and NGF.

### 1.3. Poor Blood-Brain Barrier Permeability

Attempts to use BDNF and NGF as therapeutics for central nervous system (CNS) disorders typically utilize central administration routes that bypass the BBB, including intracerebroventricular (ICV) injection, intraparenchymal injection, and intranasal administration [11]. This is largely due to their severe limitation in crossing the blood-brain barrier; however, challenges related to direct central administration still remain [11]. Intracerebroventricular and intraparenchymal routes of administration are highly invasive. Although intranasal administration is noninvasive, it generally results in lower efficiency of drug delivery to brain tissues as nasal mucosa can inhibit molecule permeability which is compounded by a lack of literature on appropriate nasal delivery [15]. The lack of methods for efficient and noninvasive delivery of BDNF and NGF to the brain has therefore presented a roadblock to studying the direct administration of these neurotrophins. To circumvent this issue, there have been some studies involving indirect modification of the levels of neurotrophins, specifically BDNF.

Indirect modification is achieved by the administration of therapeutics that elicit an increase in neurotrophins in the CNS, including drugs classified as NMDA receptor antagonists, cholinesterase inhibitors, statins, and sigma-1 receptor agonists. NMDA receptor antagonists, including memantine, ketamine, and dextromethorphan, have been used in humans as experimental therapeutics for stroke. NMDA receptor antagonism is a mechanism of action in several Alzheimer's disease therapeutics, including memantine. In addition to NMDA receptor antagonism, memantine was found to increase BDNF levels in macaques, as measured by upregulated mRNA and protein expression of BDNF [16]. Because of this impact on neurotrophic factor expression, memantine and other NMDA receptor antagonists are being studied as potential stroke therapeutics in animal models as well as in human clinical trials. One completed trial investigating memantine as a therapeutic for poststroke aphasia showed that memantine treatment resulted in an improvement in speech compared to placebo but did not measure BDNF levels, so it is unclear what mechanisms underlie the benefits to speech associated with memantine treatment [17]. In mice, memantine resulted in increased BDNF signaling, a reduction in reactive astrogliosis, improved vascularization, and improvements in functional recovery [18]. In addition to memantine, several other therapeutics have been shown to modify BDNF levels. Donepezil, a cholinesterase inhibitor used for the treatment of Alzheimer's disease, increased serum BDNF in Alzheimer's disease patients [19], while atorvastatin, a HMG-CoA reductase inhibitor, increased serum BDNF levels and improved functional recovery following stroke [20]. Sigma-1 agonists, which activate TrkB receptors, have been shown to increase BDNF levels in the rat hippocampus [21] and demonstrate neuroprotective effects [22, 23] in a non-SOD1 motor neuron disease model, Huntington's disease model, and an SOD1 ALS model through the ERK and Akt pathways downstream of TrkB [24–26].

Stem cell therapy is another method currently being studied for its potential to increase BDNF levels. *In vitro* studies have shown that neural progenitor cells are capable of releasing neurotrophins including BDNF, NGF, and NT-3 [21]. Further preclinical studies have demonstrated the utility of stem cells to elevate BDNF in models of neurological disorders. Implantation of neural stem cells yielded elevated BDNF and increased synaptic density in a mouse model of Alzheimer's disease [22] while mouse models of ischemia reveal that administration of embryonic stem cells (ESCs) can lead to the restoration of behavioral deficits, synaptic connections, and damaged neurons through the release of neurotrophic factors such as BDNF, NGF, and GDNF [23–25].

### 1.4. Lack of Consensus on Measurable Outcomes of Recovery

Several case studies examining the administration of neurotrophic factors, specifically NGF, were published during the 1980s and 1990s but resulted in a lack of consensus on measurable outcomes of recovery. Trials involving central administration of NGF to aid recovery after cerebral ischemia were preceded by its use in Parkinson's disease and Alzheimer's disease [26]. Clinical use of exogenous neurotrophic factors was later examined in two case studies examining the impact of NGF administered via intracerebroventricular infusion on the recovery of infants following a hypoxic/ischemic event. Results of this intervention demonstrated measurable improvements in cognitive and motor performance, including improvements in cerebral perfusion and Glasgow coma score, among other measurements [27]. However, it is difficult to draw any generalized conclusions regarding the efficacy of centrally administered neurotrophins in humans given that these results represented only several individuals within case studies.

Many of the ongoing clinical studies are aimed at using rehabilitation methods such as exercise, transcranial magnetic stimulation (TMS), or hyperbaric oxygen to increase neurotrophin levels, specifically BDNF, and NGF (Table 1). An earlier clinical trial demonstrated increased BDNF levels within the cerebrospinal fluid following intrathecal administration of recombinant BDNF in patients with amyotrophic lateral sclerosis (ALS) [28]. While patients in this clinical trial did not experience serious or painful side effects due to administration, results failed to improve outcome measures. However, a subgroup of patients with severely impaired respiratory problems did significantly improve when compared to placebo groups. This clinical trial demonstrates the possibility of using recombinant BDNF and other neurotrophins to improve severe neurological conditions as other trials involving subcutaneous and intrathecal administration of BDNF are in progress for other conditions such as ALS and spinal cord injury [29].

### 1.5. The Impact of BDNF Polymorphisms

The beneficial impact of treatment effectiveness is further limited by the presence of BDNF polymorphisms. One of the more studied genetic variants of BDNF is the *val66met* single nucleotide polymorphism (SNP), which is common in humans, particularly in Asian (40-50%) and Caucasian (25-32%) populations [30]. The *val66met* SNP is linked to a decrease in activity-dependent BDNF release (but not constitutive BDNF release) and reduced cortical plasticity [31]. It is possible to track and compare the recovery and outcomes of ischemic stroke patients with the normal or abnormal genetic variants of the neurotrophic factor to better understand the effects of neurotrophic factors in cognitive and motor recovery. Although it is debated, it has been suggested that *val66met* polymorphism may play a role in neurological and neuropsychiatric disorders. The inconsistency in results is complicated by variations in the genetic model used, age, sex, ethnicity, and other factors [32]. Furthermore, the implications of this polymorphism in stroke recovery are not well defined. Recovery of stroke patients has been tracked in several studies investigating cognitive and motor function. The results have been mixed, with some studies suggesting that this polymorphism is linked to worse outcomes compared to the normal BDNF gene and other studies reporting that worse outcomes were seen for short-term but not long-term recovery or that there was no impact on recovery [33–36].

### 1.6. Ongoing Clinical Trials for Stroke

There is currently only one clinical trial evaluating central administration of exogenous neurotrophic factors (Table 2). This trial, designated NCT03686163, has recruited 106 participants to evaluate the effects of intranasal NGF for acute ischemic stroke (20 *μ*g/day) for two weeks beginning at least 72 hours poststroke. Results are expected in late 2020. Additionally, there are several ongoing or recruiting trials seeking to evaluate the potential to enhance stroke recovery of therapeutics that can influence BDNF levels in the CNS, including memantine for enhanced stroke recovery (NCT02144584) and evaluation of memantine vs. placebo on ischemic stroke outcome (NCT02535611) as well as use of donepezil in combination with transcranial direct current stimulation and intensive speech therapy (NCT04134416). Given the abundance of preclinical research using BDNF and BDNF-enhancing therapeutics, it is notable that BDNF itself is not used as a potential stroke therapeutic in current clinical trials. This may be partially due to its severe limitation in crossing the blood-brain barrier and the resulting challenges related to drug delivery and direct central administration in humans [11] whereas several FDA-approved small molecule therapeutics that cross the blood-brain barrier have been shown to elicit increases in BDNF, including memantine, donepezil, and atorvastatin, which may present a more attractive clinical option. In addition to pharmacologic interventions, there are also interventions involving exercise or motor therapy to attempt to increase endogenous neurotrophin levels.

### 1.7. Traumatic Brain Injury

Although administration of neurotrophic factors to treat stroke in a clinical setting has not been a primary focus of recent literature, there is substantial interest in understanding the role that endogenous neurotrophic factors play in recovery following other forms of injury such as TBI to optimize recovery. TBI occurs following a bump, blow, or jolt to the brain that causes brain edema and results in neuronal cell death. Treatment options for TBI are similarly lacking, with typical immediate interventions including hyperosmolar therapy to relieve intracranial pressure [37, 38] and invasive decompressive surgery [39]. Although there are a variety of pharmacological interventions that can be used following TBI depending on the severity and details of the brain injury, many of these are used to manage TBI sequelae including seizure, clotting, depression, and anxiety as opposed to enhancing neuroprotection and neural repair mechanisms to address cell death. Preclinical and clinical TBI studies focused on the pathologies can be further exacerbated by severe secondary damage, which is driven by an increased inflammatory response as well as a relatively hypoxic environment [37, 38, 40] and invasive decompressive surgery [39]. Although there are a variety of pharmacological interventions that can be used following TBI depending on the severity and details of the brain injury, many of these are used to manage TBI sequelae including seizure, clotting, depression, and anxiety as opposed to enhancing neuroprotection and neural repair mechanisms to address cell death (Table 2).

The direct administration of BDNF as a therapeutic option post-TBI has centered around the role of BDNF in promoting an anti-inflammatory cytokine milieu. In fact, BDNF has been implicated in downregulating the inflammatory response in other pathological states besides TBI/stroke. For example, in female rats inoculated with Streptococcus pneumoniae meningitis, intracisternal BDNF infusions were associated with a significant decrease in inflammatory cytokines, including IL-1B, TNF-a, and NF-*κ*B, and moreover, this response was inhibited when TrkB receptor inhibitor was coadministered [41]. To specifically investigate the anti-inflammatory effects of BDNF on a TBI animal model, Yin et al. attached a collagen-binding domain (CBD) onto BDNF and delivered this combination intracerebroventricularly, as a mechanism to improve BDNF bioavailability [42]. It was revealed that BDNF-CBD was associated with a decrease in brain edema, a reduced amount of NF-*κ*B, and an increased expression of TrkB post-TBI. Further, these effects were reversed via the administration of a TrkB receptor antagonist. To address BDNF's difficulty in crossing the BBB, Kim et al. injected BDNF-filled nanoparticles via IV three hours post-TBI injury in mice [35]. This resulted in a significantly increased level of brain BDNF that correlated with an improved neurological severity score (NSS) test in these mice.

Similar to BDNF, NGF has also been studied in the context of taming the post-TBI inflammatory response as well as its neuroprotective effects. Many TBI research studies focus on the role NGF plays in synaptic transmission, by promoting a more anti-inflammatory cytokine milieu. In an *in vitro* study, NGF attenuated the robust proinflammatory response to LPS-induced monocytes significantly decreasing NF-*κ*B, IL-1b, and IL-6 mRNA levels [43]. In a similar study, Chiaretti et al. targeted cerebrospinal fluid (CSF) from children immediately following severe TBI and 48 hours after injury [44]. It was discovered that NGF concentrations in cerebrospinal fluid are a biological marker of brain damage following TBIs. The upregulation of NGF within the first 48 hours of injury, when paired with lower IL-1b expression, presents a favorable neurological outcome [44]. These papers illustrate the role NGF has in decreasing inflammation after TBI. Furthermore, these preclinical studies create a foundation for the creation and improvement of clinical trial drug therapies for TBI patients. In 2017, this same research group delivered NGF intranasally to children with severe TBI and found that there was significant cognitive improvement, cerebral perfusion, and brain glucose metabolism associated with the therapy [44].

## 2. Conclusion

Determining the role of BDNF and NGF in stroke and TBI is complex and often contradictory. While animal and *in vitro* studies demonstrate the potential of these two neurotrophins to improve recovery, the lack of clinical research and the lack of consensus among clinical research outcomes emphasize the gap in the translation of BDNF and NGF research from bench to clinic. From these studies, it is clear that neurotrophins, specifically BDNF and NGF, administered directly and indirectly have a growing role in increasing neurogenesis and functional recovery after stroke and TBI and that BDNF and NGF have a synergistic role in motor learning and cognitive recovery. Bearing these partial successes in mind, the lack of clinical research due to the ineffectiveness of treatment options serves as an impetus for the necessity of further investigation not only to identify but also to resolve the roadblocks of the translation of this research.

## Figures and Tables

**Table 1:** Impact of neurotrophins on outcome measures in human stroke.

Stroke and neurotrophins						
Study	Dates of study	Number of participants and sex	Age (years)	Inclusion criteria	Treatment administered	Outcome measures
Exogenous administered treatment						
The neurotrophic effects of lithium carbonate following stroke: a feasibility study	2010-2017	12, all	≥40	Age, English speaking, stroke within 12 months	Lithium carbonate, 0.4-0.8 mmol/L for 2 months	Increase in total brain gray matter volumes, cognitive tasks of the neurological disorders and stroke, serum BDNF levels, serum lithium and creatinine levels
Kinetics of plasma and serum levels of BDNF in patients with ischemic stroke	2011-2012	50, all	≥18	Age, recent ischemic stroke, informed consent, cerebral imaging	Intravenous fibrinolysis using rt-PA to increase circulating BDNF	Measurement of plasma levels of BDNF Measurement of serum levels of BDNF
The STem Cell Application Researches and Trials In NeuroloGy-2 (STARTING-2) Study	2012-2017	60, all	30-75	Stroke within 90 days, radiological legions, neurological deficits, informed consent	Mesenchymal stem cell intravenous transplantation	Categorical shift in mRS, cognitive battery, exploration of biomarkers SDF-1*α* (chemokine) S100*β* (protection and regeneration), HIF-1 (preconditioning), circulating MSCs and MSC-derived microparticles CD105-CXCR4-PS BDNF levels and its polymorphism, and VEGF levels
Effects of intranasal NGF for acute ischemic stroke	2016-2020	106, all	≥18	Age, acute ischemic stroke, informed consent	Intranasal NGF 20 *μ*g/d for 2 weeks	Neurological function (low mRS score)
Study the result of ayurvedic SUVED & Reimmugen (colostrum) treatment on vascular disease, CAD, CVA, DVT	2016-2017	96, all	18-70	Diagnosis of vascular disease leading to IHD, CAD, CVA, DVT, PAD at any stage	SUVED ayurvedic formulation in Ghana (concentrated) in capsules; 500 mg each, Reimmugen, whole cow colostrum in powder put in capsules; 300 mg each	Changes in IMT as an indicator of atherosclerosis reversal, assessing the development/risk of ischemic events in other circulations
Brain correlates of multimodal rehabilitation in chronic poststroke aphasia	2019-2020	20, all	≥18	Age, diagnosis of poststroke aphasia	5 mg and 10 mg donepezil tablet, intensive language action therapy, transcranial direct current stimulation	Western Aphasia Battery Assessment, Stroke and Aphasia Quality of Life Scale 39, Communication Activity Log, Stroke Aphasia Depression Questionnaire
Evaluation of Memantine Versus Placebo on Ischemic Stroke Outcome (EMISO)	2015-2017	47, all	≥18	age, confirmation of ischemic stroke in MCA territory by imaging, presentation at first 24 hrs of disease onset	20 mg/d (2 tab 5 mg) memantine for 7 days and then 10 mg/d (1 tab 5 mg) memantine for 21 days or placebo tablet for 21 days	Changes in neurological deficit by National Institute of Health Scale Score (NIHSS), assessed disability by modified Rankin scale (mRS)
Memantine for enhanced stroke recovery	2014-2022	20, all	≥18	Age, diagnosis of ischemic stroke, arm weakness, ability to swallow pills, supratentorial location of stroke, living independently prior to stroke, able to voluntarily move affected UE	Memantine or placebo treatment given increasing by 7 mg (1 capsule) per week until a goal dose of 28 mg daily (goal dose) for 90 days	Motor Activity Log, ten-meter walk test, Stroke Impact Scale (SIS), Cancellation Tests, Grip Strength Test, Montreal Cognitive Assessment
Brain stimulation						
Use of deep transcranial magnetic stimulation after stroke	2010-2014	15, all	18-85	Age, acute ischemic stroke, neurological deficits after stroke, informed consent, NIHSS ≤ 18	Deep TMS (transcranial magnetic stimulation 10 Hz) 7; 15-minute sessions of TMS to increase secretion of BDNF	mRS < 2 and BI > 95 obtained at 3 months after stroke onset, safety, neurological outcome assessed by NIHSS at discharge < 5 or showing improvement of at least 8 points from the initial stroke score or improvement of at least 2 points on item 6 of the NIHSS (motor score leg), good neurological outcome as assessed by NIHSS at 3 months < 5 or showing improvement of at least 8 points from the initial stroke score or improvement of at least 2 points on item 6 of the NIHSS (motor score leg)
IMPULSE—stimulation of brain plasticity to improve upper limb recovery after stroke	2020-2023	90, all	18-80	Age, 8 weeks-12 months after ischemic stroke, low mRS score, Action Research Arm Test (ARAT) score 13-50, both inclusive, Shoulder Abduction Finger Extension (SAFE) score ≥ 5, informed consent	Cerebrolysin 30 mL once daily (+70 mL 0.9% saline), noninvasive brain stimulation 2 mA/35 cm² for 2 × 20 minutes, once daily	ARAT, NHPT, hand grip dynamometry, NIHSS
Cortical priming to optimize gait rehabilitation in stroke: a renewal	2020-2025	100, all	18-80	Age, stroke within 3 months, residual hemiparetic gait deficits, ability to walk without ankle orthotic, walking speed lesser than 1.4 m/s, lower limb Fugl-Meyer motor score between 20 and 30, at least 5 deg of ankle dorsiflexion necessary to perform the ankle-tracking task	Transcranial direct current stimulation (tDCS) 1 mA tDCS, ankle motor training, high-intensity interval speed-based treadmill training (HIISTT)	Walking speed with 10-meter walk test, BDNF, salivary samples for BDNF, corticomotor excitability using TMS, cognitive battery
Physical activity						
Effects of upper limb motor and robotic training over neuroplasticity and function capacity	2012-2017	51, all	≥18	Stroke within 6-36 months, clinically unstable, informed consent, low upper limb Brunnstrom scale score, minimal wrist extension	ICT two times a week for ten weeks, robotic occupational therapy three times a week for twelve weeks	Change on motor function, neuroplasticity as assessed by BDNF, psychological evaluation assessed by PSS-10, corticospinal excitability as assessed by TMS, neurologic evaluation as assessed by electroencephalography
Effects of combined resistance and aerobic training vs. aerobic training on cognition and mobility following stroke	2013-2016	72, all	Child adult, older adult	Stroke, ability to walk, no pain limitation, living in community for 3 months poststroke, motor impairment, informed consent	Combined resistance and aerobic training For the group randomized to AT+RT, patients will gradually be progressed from 1 to 2 sets and then from 10 to 15 repetitions and then increase resistance	Cognitive function, body composition, biochemical changes (blood samples BDNF, IGF-I, homocysteine, and C-reactive protein), functional mobility
The safety and tolerability of an aerobic and resistance exercise program with cognitive training poststroke	2014-2019	132, all	≥18	Ischemic or hemorrhagic stroke, high mRS score, recently discharged from the hospital, less than ideal physical activity, able to walk ≥10 meters with or without assistance	ARET: combined aerobic and resistance exercise training; CTI: cognitive training intervention	Number of participants with treatment-emergent serious adverse events, adherence to a 12-week combined exercise and cognitive training protocol versus a sham group, change in cognitive performance on cognitive neuropsychological battery done at pre-, post- and 6-month follow-up visits, change in health-related quality of life–depression, change in health-related quality of life-daily activities, change in blood plasma concentration of BDNF
Aerobic trainings on stroke patients	2016-2018	23, all	20-80	Stroke, MMSE ≥ 24, no acute coronary syndrome	Aerobic exercise training	Peak CO, exercise VO_2_peak, OUES, VCO_2_ ratio Ve-VCO_2_, differences of the brain ∆[O_2_Hb], differences of the brain ∆[HHb], differences of regional blood volume ∆[THb], PCS, MCS, MMSE, BDNF levels, percentage of cell bearing neurites, neuron images
Serum BDNF role as a biomarker for stroke rehabilitation	2017-2019	150, female	≥19	Unilateral stroke, rehabilitation within 1 month of stroke onset, motor impairment	Conventional inpatient rehabilitation Comprehensive inpatient rehabilitation for 2 weeks	Serum BDNF levels, serum proBDNF, MMP-9
Effects of combined cognitive training with aerobic exercise in stroke patients with MCI	2018-2021	75, all	20-90	Ischemic or hemorrhagic stroke, age, low cognitive assessment score, cognitive impairment, ability to follow instructions, ability to exercise, ability to walk	Aerobic exercise training, computerized cognitive training	Cognitive battery, BDNF val66met genotype saliva samples, serum BDNF level, TAC, glucose indicator, plasma lipid level
Chiropractic care plus physiotherapy compared to physiotherapy alone in chronic stroke patients	2019-2019	100, all	Child, adult, older adult	Stroke within 12 weeks of trial, neurological deficits, upper/lower limb weakness, Fugl-Meyer Assessment (FMA) motor score of less than 80 at the time of enrollment	Chiropractic care	FMA, stroke-specific quality of life scale, mRS, TUG, HRV, daily movement, blood marker BDNF, blood marker GDNF, blood marker IGF2, transcranial magnetic stimulation
Biologic mechanisms of early exercise after intracerebral hemorrhage	2019-2021	40, all	≥18	Supratentorial intracerebral hemorrhage with or without intraventricular hemorrhage, premorbid mRS score 0-2, informed consent	Supine cycle ergometry of the lower extremities	Change in interleukin-1beta level in blood, change in interleukin-6 level in blood, change in tumor necrosis factor-alpha level in blood, change in C-reactive protein level in blood, change in BDNF level in blood, change in interleukin-1beta level in CSF, change in interleukin-6 level in CSF
Group Lifestyle Balance™ for individuals with stroke (GLB-CVA)	2019-2021	65, all	18-65	Age, BMI ≥ 25, stroke within 12 months, physician approval	GLB weight loss intervention, Group Lifestyle Balance	Change in weight, biomarker analysis (isrin, angiogenic factors VEGF, total homocysteine, Lp-PLA2, ICF-1, BDNF, and tau proteins, physical activity, blood pressure, cholesterol)
Muscle trajectories in acute stroke patients	2019-2024	200, all	≥18	Age, hospitalized at neurology ward of UZ Brussel, stroke, informed consent	Follow-up assessments	Functional ambulation categories, 6-minute walking test, circulating biomarkers, blood sampling circulating biomarkers: brain-derived neurotrophic factor (BDNF), inflammation-related biomarkers
Rehabilomics study in stroke patients after robotic rehabilitation	2020-2021	100, all	55-85	Stroke within 2-24 weeks, age, ability to perform rehabilitation treatment, language abilities	Robotic-assisted intervention (30 sessions, 5 times a week)	Presence/absence of rs6265 in the BDNF, presence/absence of 5-HTTLPR in the SLC6A4, change in promoter methylation levels of BDNF gene, change in promoter methylation levels of SLC6A4 gene, cognitive battery
Exercise-primed upper extremity motor practice in chronic stroke	2021-2022	10, all	21-90	Unilateral stroke within 6 months, impaired shoulder flexion, arm movement impairment, passive range of motion, age, ability to exercise, ability to communicate	Aerobic exercise+DDP, 15 minutes of aerobic exercise on a recumbent stationary cycle, 200 repetitions on an upper extremity rehabilitation game called DDP	Change in upper extremity impairment as assessed by the FMA extremity, change in upper extremity as assessed by the Wolf Motor Function Test, change in physical function and health-related quality of life as assessed by Stroke Impact Scale, change in neuroplastic potential as assessed by paired associative stimulation, assessment of BDNF
Aerobic exercise training in acute ischemic stroke	2021-2022	30, all	≥18	Age, stroke, medically stable, English speaking, ability to move lower limbs	Aerobic exercise training 5-day, power-assisted, low to moderate intensity, aerobic exercise training programme. Exercise duration to progress from 10 minutes on day 1 to 30 minutes on day 5	Safety of aerobic exercise training, acceptability of aerobic exercise training, rectus femoris cross-sectional area, rectus femoris muscle thickness, vastus lateralis muscle thickness, vastus lateralis angle of pennation, cognitive function, anxiety, depression, aerobic exercise-induced changes in mature BDNF serum and plasma
Serum and plasma analysis of BDNF						
Neuroactive steroids in acute ischemic stroke	2016-2016	80, all	60-90	age, acute ischemic stroke, 9 ≥ score on Glasgow coma scale, females in menopause, patients without prior cognitive impairment, informed consent, no prior cognitive impairment	Observed changes in plasma BDNF and nitrites	Neurological deficit, cognition, emotional state, functional dependency of daily life activities, cortisol, quantification of nitrite concentration, BDNF quantification in plasma
Functional prognosis in patients with ischemic stroke according to the therapeutic strategy used	2016-2020	300, all	≥18	Ischemic stroke, age, informed consent	A blood sample taken at different times to study the value of growth differentiation factors (GDF) 8, 11, and 15 and brain-derived neurotrophic factor as prognostic biomarkers	Rate of handicap, serum levels of biomarkers of stress
Effects of repetitive hyperbaric oxygen therapy in patients with acute ischemic stroke	2018-2020	60, all	18-80	Acute ischemic stroke, Glasgow coma scale more than 10	Hyperbaric oxygen, 10 sessions of HBOT at 2.0 atmosphere absolute (ATA) for one hour in a hyperbaric chamber pressured with compressed air to upregulate expression of glial-derived neurotrophic factor (GDNF) and nerve growth factor (NGF)	Change in National Institutes of Health stroke score before and after treatment with hyperbaric oxygen therapy, hospital mortality, hospital length of stay
Effect of lifestyle changes on BDNF level after stroke	2018-2019	12, all	30-90	History of stroke, ability to move at least 10 feet with little assistance, ability to travel to intervention site	Assessing BDNF levels at different time points throughout study	BDNF level–final, BDNF level-postexercise, BDNF genotype, cardiovascular fitness-VO_2_ max, cardiovascular fitness–METs, 6-minute walk test
Role of genetic polymorphism in neuroplasticity involved in dysphagia recovery	2018-2019	220, all	Child, adult, older adult	Lesions from stroke and TBI, patients hospitalized for 30 days and were followed up at 3 months after lesion, informed consent, patients able to swallow	Blood serum analysis	Change in FOIS, change in BBS, change in MRC grade disability level, cognitive battery, blood serum analysis
White matter integrity according to BDNF genotype after stroke	2018-2019	58, all	18-80	Diagnosed with first-ever hemispheric ischemic infarction with damage to the supratentorial area confirmed by brain MRI within 2 weeks after stroke onset	BDNF serum analysis	Changes in FA in CST, the intrahemispheric corticocortical tract from the M1PMv and CC from 2 weeks to 3 months after stroke according to BDNF genotype. BDNF genotype SNP: Met substitution for Val at codon 66 (Val66Met; rs6265)
Moderate intensity aerobic training in subacute and chronic stroke patients-the influence on BDNF and upper-limb rehabilitation. A protocol for a randomized control trial and health economic evaluation	2019-2020	30, all	≥18	Stroke within the last 3 months or more, ability to move shoulders	Assessing BDNF levels at different time points throughout study	BDNF serum levels, ARAT, the FMA, 10-meter walking test, trunk sway in standing with eyes closed, cognitive battery, the FSS, stroke impact scale
Muscle trajectories in acute stroke patients	2019-2024	200, all	≥18	Age, hospitalized at neurology ward of UZ Brussel, stroke, informed consent	Follow-up assessments	Functional ambulation categories, 6-minute walking test, circulating biomarkers, blood sampling circulating biomarkers: brain-derived neurotrophic factor (BDNF), inflammation-related biomarkers

**Table 2:** Impact of neurotrophins on outcome measures in human TBI.

Study	Dates of study	Number of participants and sex	Age (years)	Inclusion criteria	Treatment administered	Outcome measures
Exogenously administered treatment						
Nerve growth factor for TBI	2010-2017	106, all	18-65	Age, moderate to severe TBI	Intranasal NGF 20 *μ*g/d for 2 weeks	GOS, mRS, BI, HAMA, HAMD
Cerebrolysin neural repair therapy in children with TBI and cerebral palsy	2014-2016	100, all	3 months-18 yrs	Cerebral palsy with mental retardation, severe perinatal brain insult	NGF cerebrolysin	Neurodevelopment: IQ assessment at baseline and after 3 and 6 months of therapy
Derivatives of omega-3 HUFA as biomarkers of TBI	2017-2023	45, all	18-55	Age, verified TBI, ability to swallow, not pregnant, English speaking, informed consent, coenrolled in PARC-TBI protocol or TRACK-TBI, GCS 3-15	1, 1000 mg/day n-3 HUFA, or 2, 4,000 mg/day n-3 HUFA within 24 hours of injury for 14 days	Relationship of varying doses of n-3 HUFAs on blood levels of the following bioactive metabolites indicators of neuroinflammatory damage including BDNF, relationship of n-3 HUFA blood levels and clinical outcomes measured by the GOSE, evaluate potential adverse events
Simvastatin for mTBI	2013-2017	6, all	≥21	Age, documented hazardous duty in Iraq and or Afghanistan with the U.S. armed forces. mTBI according to American Congress of Rehabilitation Medicine (ACRM) criteria. More than 6 months since last blast trauma exposure, adequate English language skills, vision, and hearing). Elevated cholesterol levels. No use of statins during the previous year and recently. No clinically significant laboratory abnormalities (electrolytes, Body Mass Index (BMI) between 18 and 36 inclusive)	Simvastatin 40 mg/day for 12 months	CSF tau concentration, CSF BDNF
OPTIMA-TBI pilot study	2017-2021	75, all	18-65	Evidence of TBI or mTBI	Omega-3 polyunsaturated fatty acid, 6 g DHA+EPA for one month followed by 1.2 g DHA+EPA for two months. Capsules contain fish oil 1000 mg (contains 500 mg DHA & 100 mg EPA)	Biomarker endpoints (NFL), biomarker endpoint (inflammation), biomarker endpoint (neurogenesis serum levels of BDNF, delayed functional recovery, moderate/severe postconcussive symptoms, cognitive impairment)
Brain stimulation						
rTMS to improve cognitive function in TBI	2014-2019	33, all	20-65	Age, veteran, history of TBI, obtain motor threshold, stable environment, ability to attend appointments, not pregnant	Active rTMS; 20 sessions of rTMS	TMT part B, sustained improvement on executive function, change in QOL scale, moderators of response: PTSD score, treatment-induced change in functional connectivity, change in a mediator of response: BDNF
Physical activity						
Effects of aerobic exercise on cognition, mood, and fatigue following TBI	2007-2021	154, all	≥18	TBI within 6 months or more, basic mobility, English speaking	Behavioral exercise, 50 minutes of aerobic exercise on a treadmill 3 days a week for 8-16 weeks	HVLT-R, TMT A and B, digit span subtests of the WAIS-III, WCST, COWAT Stroop Word Color Test GF Index, BDI-II, blood draws for assessment of BDNF and VEGF levels
Microvascular injury and BBB dysfunction as novel biomarkers and targets for treatment in TBI	2017-2020	120, all	18-85	Age, evidence of TBI		Change in brain volume with BBB dysfunction, change in serum biomarkers BBB dysfunction vWF, BDNF, GFAP, S100*β*, sTau, and sNFL, changes in GOS-E, change in RPSQ, change in PROMIS, change in posttraumatic epilepsy
Aerobic exercise and cognitive training effects on postconcussive symptomology	2018-2019	34, all	≥18	TBI, ability to exercise, persistent symptoms, access to smartphone	30 minutes of aerobic exercise followed by a 20-minute cognitive training (CT) program	The Rivermead Post Concussion Symptoms Questionnaire, NIH Toolbox Cognition Battery-working memory, NIH toolbox Cognition Battery-attention
Treating persistent postconcussion symptoms with exercise	2019-2021	58, all	18-65	mTBI, cleared for physical activity, low risk for cardiopulmonary disease, exercise intolerance (inability to exercise at preinjury intensity/duration due to acute presentation of symptoms)	Aerobic Exercise Protocol (AEP) exercise 20 minutes per day or until symptom exacerbation, 5-6 days per week	Change in symptom burden, change in sleep duration, change in daytime sleepiness, change in BDNF, change in cytokine profile, change in TL, change in fatigue. Change in anxiety, change in function related to headaches, change in depression, MRS quantification of GABA/glutathione
Serum/sample analysis						
S100B in intensive care patients with and without TBI	2007-2019	600, all	≥18	Patients of the Department of Neurosurgery, University of Erlangen Nürnberg, TBI patients, intracranial tumor patients, intensive care patients, informed consent	Blood, cerebrospinal fluid, and urine samples, in all subjects, blood (4 mL), cerebrospinal fluid (4 mL), and urine (4 mL) samples were collected daily as part of the clinical routine at 6:00 AM	GOS, Karnofsky performance status score
Microvascular injury and BBB dysfunction as novel biomarkers and targets for treatment in TBI	2017-2020	120, all	18-85	Age, evidence of TBI		Change in brain volume with BBB dysfunction, change in serum biomarkers BBB dysfunction vWF, BDNF, GFAP, S100*β*, sTau, and sNFL, changes in GOS-E, change in RPSQ, change in PROMIS, change in posttraumatic epilepsy
Epigenetic effects on TBI recovery	2017-2023	300, all	3-18	TBI, orthopedic injury	Blood and saliva biosamples are collected at all time points and CSF when available acutely for epigenetic and proteomic analysis of BDNF	NIHTB-CB, BRIEF-2 or BRIEF-P, strengths and difficulties questionnaire, and Vineland Adaptive Behavior Scales, Third Edition (Vineland-3) (6, 12 months' postinjury)

mRS: modified Rankin score; BI: Barthel index; PSS-10: Perceived Stress Scale; TMS: transcranial magnetic stimulation; BDNF: brain-derived neurotrophic factor; SDF1*α*: stromal cell-derived factor 1*α*; CST: corticospinal tract; M1PMv: ventral premotor cortex; CC: corpus callosum; HIF: hypoxia-inducible factor-1; VEGF: vascular endothelial growth factor; IGF-1: insulin-like growth factor I; FA: fractional anisotropy; DDP: Duck Duck Punch; CO: peak cardiac output; VO_2_peak: peak exercise oxygen consumption; OUES: oxygen uptake efficiency slope; Ve-VCO_2_: ventilation/VCO_2_ ratio; ∆[O_2_Hb]: differences of the brain tissue oxyhemoglobin; ∆[HHb]: differences of the brain tissue deoxygenation; ∆[THb]: differences of regional blood volume; PCS: physical component score; MCS: mental component score; MMSE: minimental status examination; TAC: total antioxidant capacity; FOIS: Functional Oral Intake Scale; BBS: Berg Balance Scale; MRC: Medical Research Council; SNP: single nucleotide polymorphism; Met: a methionine; Val: valine; ARAT: Action Research Arm Test; FMA: The Fugl-Meyer Assessment-Upper Extremity Scale; FSS: Fatigue Severity Scale; TUG: Timed up and Go Test; HRV: Heart Rate Variability; GDNF: glial cell-derived neurotrophic factor; IGF2: insulin-like growth factor 2; mBI: Modified Barthel Index; CSF: cerebrospinal fluid; Lp-PLA2: lipoprotein-associated phospholipase A2; NHPT; Nine-Hole Peg Test; NIHSS: National Institutes of Health Stroke Scale; HVLT-R: Hopkins Verbal Learning Test-Revised; TMT: Trail Making Tests A and B; digit span subtests of the WAIS-III; WCST: Wisconsin card sort test; COWAT: Controlled Oral Word Association Test; GF: Stroop Word Color Test Global Fatigue Index; BDI-II: Beck Depression Inventory-II; GOS: Glascow Outcome Score; HAMA: Hamilton Anxiety Scale; HAMD: Hamilton Depression Scale; IQ: neurodevelopment: intelligence quotient; QOL: quality of life; NIHTB-CB: NIH Toolbox Cognition Battery; BRIEF-2: Behavior Rating Inventory of Executive Function, Second Edition; BRIEF-P: Behavior Rating Inventory of Executive Function, Preschool Version; Vineland-3: Vineland Adaptive Behavior Scales, Third Edition; GOSE: Glasgow Outcome Scale-Extended; BBB: blood-brain barrier dysfunction (vWF, BDNF, GFAP, S100*β*, sTau, and sNFL); RPSQ: Change in Rivermead Postconcussion Symptom Questionnaire; PROMIS: Change in Patient-Reported Outcomes Measurement Information System; NFL: change in posttraumatic epilepsy biomarker endpoints; TL: telomere length; ICT: Induced Constraint Therapy; MMP-9: matrix metallopeptidase 9; CD105-CXCR4: C-X-C chemokine receptor type 4-PS (phosphoserine).
